# Isolation of Liver Aldehyde Oxidase Containing Fractions from Different Animals and Determination of Kinetic Parameters for Benzaldehyde

**DOI:** 10.4103/0250-474X.40337

**Published:** 2008

**Authors:** R. S. Kadam, K. R. Iyer

**Affiliations:** Department of Pharmaceutical Chemistry, Bombay College of Pharmacy, Kalina, Mumbai - 400 098, India

**Keywords:** Rabbit, guinea pig, liver aldehyde oxidase, hepatic, benzaldehyde

## Abstract

Aldehyde oxidase activity containing fractions from rabbit, guinea pig, rat and mouse livers were obtained by heat treatment and ammonium sulfate precipitation. Aldehyde oxidase activity was observed in rabbit and guinea pig livers, while aldehyde oxidase activity was absent in rat and mouse liver fractions. Enzyme kinetic parameters, K_m_ and V_max_, were determined for the oxidation of benzaldehyde to benzoic acid by rabbit and guinea pig liver fractions, by spectrophotometric method, with potassium ferricyanide as the electron acceptor. The K_m_ values obtained for both animal liver fractions were in the range of 10.3-19.1 μM.

Aldehyde oxidase (E.C.1.2.3.1) (AO) belongs to the small family of molybdoflavoproteins that includes xanthine oxidoreductase (XOR) and sulphate oxidase^1^. AO is a cytosolic enzyme that contains FAD, molybdenum and iron-sulfur centers, and is closely related to xanthine oxidase. The enzyme can catalyze the oxidation of number of aldehydes and nitrogenous heterocyclic compounds. Moreover, the enzyme in presence of an electron donor can also mediate the concomitant reduction of a variety of compound such as sulfoxides, N-oxides, nitrosamine, hydroxamic acids, azo dyes, oximes, epoxides, aromatic nitro compounds and 1,2-benzisoxazole derivatives^1^. AO possesses wider substrate specificity as compared to xanthine oxidase and plays an important role in the metabolism of xenobiotics as well as endobiotics^2^. AO is an important Phase I oxidative enzyme that contributes to the metabolism of heterocyclic structures that possess an electropositive carbon centre (generally C adjacent to a heteroatom like N, S, or O)^3^. Recently, AO has been suggested to play an important role in visual processes, since the enzyme catalyzes the biotransformation of the principal component of the visual pigment *all trans*-retinal to retinoic acid, the active form of vitamin A^4^. Mammalian liver AO is also responsible for metabolism of nicotine metabolite (S)-nicotine Δ^1^,^5^-iminium ion, to (S)-cotinine in rat; the α-N-oxidation of brominidine to its 2, 3-dioxoderivatives, major metabolites in humans^4^. The metabolism of tamoxifen 4-aldehyde to the corresponding carboxylic acid in rat, activation of pyrimidine prodrug nucleus to uralic acid in mouse, rabbit and rat and the conversion of famciclovir to penciclovir in humans are all catalyzed by AO^4^.

Our laboratory has been involved in the establishment of both enzyme fraction isolation protocols and enzyme assay protocols for various important drug metabolizing enzymes. The present study was initiated for isolation of crude fraction of AO from various animal species and determination of kinetic parameters of AO for substrate benzaldehyde by spectrophotometric method.

Animal livers were obtained from the Department of Pharmacology, Bombay College of Pharmacy, Mumbai. The animals and the strains used were as follows; rat (Charles Foster), mouse (Swiss Albino), guinea pig (Albino) and rabbit (New Zealand). The animals used in this study were those that were sacrificed as part of other experiments approved by the Institutional Animal Ethics Committee. It should be noted that the potential of these experiments to alter liver function (and consequently AO content) although a possibility, was not taken into consideration since the intention of this study was to only evaluate the AO isolation procedure and AO activity determination. The livers obtained were either used fresh or stored at −70° until use. Potassium dihydrogen orthophosphate, dipotassium hydrogen orthophosphate, benzaldehyde and EDTA were purchased from S. D. Fine Chem. Ltd., Mumbai. Bradford's macro method protein estimation kit was purchased from Bangalore Genie Pvt. Ltd., Bangalore. Ammonium sulphate was purchased from Himedia Lab., Mumbai. All other chemicals and reagents used in the study were of AR grade.

Crude fractions of AO were isolated from animal livers by previously reported procedures^5^,^6^. Briefly, 10 g of liver was homogenized in 5 volume of 0.05 M potassium phosphate buffer, pH 6.8, for 5 min in a Potter glass homogenizer equipped with a Teflon pestle. The homogenate was then rapidly heated to 55^°^ on a water bath, maintained at this temperature for 5 min, and then cooled quickly to below 10^°^ in an ice bath. During both the heating and cooling steps the homogenate was stirred. The heat-treated and cooled homogenate was centrifuged at 16 000×g for 15 min and the precipitate discarded. Solid ammonium sulphate was added to the supernatant to a final concentration of 60% saturation (37.56 g/100 ml), the mixture centrifuged at 20 000×g for 15 min and the pellet was suspended in 10 ml of 0.05 M potassium phosphate buffer, pH 7.8, containing 0.005% EDTA and stored at −70° for further use. Three different samples of AO fractions were obtained from different liver samples of each animal and all of the subsequent estimations were done in duplicate for each of the three isolated AO fractions per animal.

AO activity assay was performed according to the reported method using ‘blank reversal technique’^5^. A Shimadzu spectrophotometer (UV 160 A) with matched 10 mm quartz cuvettes was used for the assay. The assay mixture contained 50 mM potassium phosphate buffer, pH 7.8, containing 0.005% EDTA, 300 μl of 0.01 M potassium ferricyanide solution, 50 μl of 0.3 M benzaldehyde and 50 μl of enzyme sample in final volume of 3 ml, in a 10 mm quartz cuvette. The blank cuvette consisted of all the components listed above except enzyme. AO activity determination was initiated by addition of enzyme and was monitored by analyzing the amount of potassium ferricyanide reduced at 420 nm at ambient temperature. The velocity of reaction was determined as μmol of potassium ferricyanide reduced per three ml per min using extinction coefficient (ε) of 2080 M^−1^ cm^−1^. One unit of enzyme activity was defined as the amount of enzyme that catalyzed the reduction of 1 μmol of potassium ferricyanide per min under the stated conditions^7^.

Protein concentrations of partially purified AO fractions were determined spectrophotometrically according to the Bradford method with Bradford macro method kit, using bovine serum albumin as standard, according to the manufacturer's instructions.

K_m_ (Michaelis-Menten constant) and V_max_ (maximum velocity) values for the oxidation of benzaldehyde to benzoic acid with rabbit and guinea pig liver AO fractions were determined^6^. Initial experiments to determine linearity of reaction with respect to both enzyme amount and time were conducted (data not shown). Product formation was kept to 10% or below for adherence to Michaelis-Menten assumptions. The assay methodology used was similar to described above for enzyme assay, except that different concentrations of benzaldehyde i.e. 5, 10, 15, 20, 25, 50, 100, and 500 μM were used. The velocity of reaction was determined as μmol of potassium ferricyanide reduced per ml per min using extinction coefficient (ε) of 2080 M^−1^ cm^−1^. K_m_ and V_max_ values were determined using the Lineweaver-Burk, Eadie-Hofstee and Hanes plotting methods. The line of best fit through the points on the plot was determined using linear regression by least squares method using Microsoft Excel (Microsoft Office XP).

Unit activity and specific activity of AO fractions from different animal liver cytosolic fractions were measured spectrophotometrically, using benzaldehyde as the specific probe substrate for AO. The mean unit AO activities and specific AO activities are listed in Table 1. AO activity was present in rabbit and guinea pig isolated liver AO fractions. AO activity was however absent in rat and mouse liver AO fractions. Further, enzyme kinetic parameter estimations were therefore done only with rabbit and guinea pig liver AO fractions. K_m_ and V_max_ values for the oxidation of benzaldehyde to benzoic acid by aldehyde oxidase fractions were measured for rabbit and guinea pig liver AO liver fractions. Mean values of K_m_ and V_max_ of AO as determined using three different plotting methods viz. Lineweaver-Burk plot, Eadie-Hofstee plot and Hanes plot are listed in Table 2.

**TABLE 1:** UNIT ACTIVITY AND SPECIFIC ACTIVITY OF AO IN RABBIT, GUINEA PIG, RAT AND MOUSE LIVER FRACTIONS

Animal liver	Mean unit activity (units/ml of enzyme solution)	Mean specific activity (units/mg protein)
Rabbit	0.284 ± 0.040	0.087 ± 0.006
Guinea pig	1.51 ± 0.173	0.613 ± 0.311
Rat	Absent	Absent
Mouse	Absent	Absent

One unit of enzyme activity was defined as the amount of enzyme that catalyzed the reduction of 1 μmol of potassium ferricyanide per min under the stated conditions, when benzaldehyde was used as the substrate. The values listed are the mean values ± standard deviation, obtained from three liver fractions for each animal, each experiment being conducted in duplicate

**TABLE 2:** VALUES OF K_M_ AND V_MAX_ OF AO BY UV USING LB, EH, AND HANES PLOTS

Animal	LB plot		EH plot		Hanes plot	
			
	K_m_	V_max_	K_m_	V_max_	K_m_	V_max_
Rabbit	14.7 ± 4.51	0.028 ± 0.005	10.3 ± 3.26	0.024 ± 0.004	10.7 ± 3.62	0.024 ± 0.003
Guinea pig	17.8 ± 7.52	0.066 ± 0.018	16.5 ± 4.53	0.060 ± 0.003	19.1 ± 1.67	0.067 ± 0.003

The values listed are the mean values ± standard deviation, obtained from three liver fractions for each animal, each experiment being conducted in duplicate. LB: Lineweaver-Burk and EH: Eadie Hofstee. K_m_ values are expressed in μM and V_max_ values are expressed in μmol of potassium ferricyanide reduced per ml per min.

In the present study, high aldehyde oxidase activity was detected in rabbit and guinea pig liver whereas, AO activity was absent in rat and mouse livers. This was expected as previous studies have indicated that little or no AO is present in rat and mouse livers^8^,^9^. Such species differences are known for AO activity, for example, high AO activity is observed in rabbit liver while AO activity is undetectable in dogs^10^.

The kinetic parameters i.e. K_m_ and V_max_ were determined using three Lineweaver-Burk plot, Eadie-Hofstee plot and Hanes plot. Of the three plotting methods, it is suggested that the Eadie-Hofstee plot is superior to the other two methods of plotting data^11^,^12^. This is due to use of low velocity values (that have most errors) without any transformation, rather than in reciprocal form as in Lineweaver-Burk and Hanes methods. Further, the Eadie-Hofstee plot is more suited to detection of both, deviation from linearity with changing substrate concentrations and detection of data of lower quality^11^,^12^. One representative guinea pig liver fraction AO kinetic parameter estimation data plotted by the three methods is shown in fig. 1. As seen in fig. 1, the superiority of EH plot is evident by the lower correlation coefficient value (0.8711) obtained as compared to the other two methods (0.9613 and 0.9992) due to the inherent stringency of this plotting method. The reported K_m_ value for benzaldehyde oxidation by AO from guinea pig and hamster liver AO is 19 μM^13^,^14^ and 11 μM for human liver AO^8^. The results obtained in this study shows that the K_m_ values as determined by Lineweaver-Burk plot, for rabbit and guinea pig liver AO fractions were 10.7 ± 3.62 μM and 19.1 ± 1.67 μM, respectively, which are in reasonable agreement with the reported values. The values of K_m_ as determined by different plotting methods are also in the same range (10.3-19.1 μM).

**Fig. 1:**
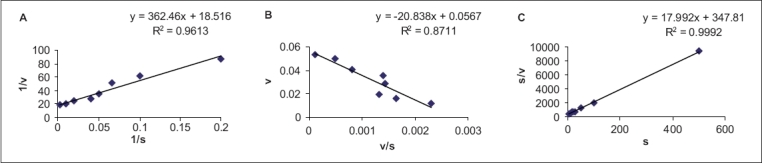
Lineweaver-Burk, Eadie-Hofstee and Hanes plots of guinea pig liver AO incubations. Guinea pig liver fractions were incubated with a range of benzaldehyde concentrations for the determination of initial velocities as indicated in the text. The top panel ‘A’ represents the Lineweaver-Burk plot, the middle panel ‘B’ represents the Eadie-Hofstee plot, and the bottom panel ‘C’ is the Hanes plot of the velocity data at different benzaldehyde concentrations. Substrate concentration [S] values are expressed in μM and initial velocity [V] values are expressed in μmol of potassium ferricyanide reduced per ml per min.

Overall, this study presents the evaluation of a simple method for the isolation of crude fractions containing AO from different animal livers and a spectrophotometric method for the determination of AO enzyme kinetic parameters in crude fractions, with benzaldehyde as the model substrate. This assay is of utility is the establishment of drug metabolism study protocols in drug metabolism research.
